# Various 3D printed materials mimic bone ultrasonographically: 3D printed models of the equine cervical articular process joints as a simulator for ultrasound guided intra-articular injections

**DOI:** 10.1371/journal.pone.0220332

**Published:** 2019-08-06

**Authors:** Alexandra Beaulieu, Alex zur Linden, John Phillips, Luis G. Arroyo, Judith Koenig, Gabrielle Monteith

**Affiliations:** 1 Department of Clinical Studies, Ontario Veterinary College, University of Guelph, Guelph, Ontario, Canada; 2 Digital Haptics Laboratory, College of Arts, University of Guelph, Guelph, Ontario, Canada; 3 Center for Advanced Manufacturing and Design Technologies, Sheridan College, Brampton, Ontario, Canada; University of Vigo, SPAIN

## Abstract

**Introduction:**

In the equine racehorse industry, reduced athletic performance due to joint injury and lameness has been extensively reviewed. Intra-articular injections of glucocorticoids are routinely used to relieve pain and inflammation associated with osteoarthritis. Intra-articular injections of pharmaceutical agents require practice for precise needle placement and to minimize complications. Training on simulators or models is a viable alternative for developing these technical skills. The purpose of this study was to compare the qualitative ultrasonographic characteristics of three-dimensional (3D) printed models of equine cervical articular process joints to that of a dissected equine cervical spine (gold standard).

**Methods:**

A randomized complete block design study was conducted in which a total of thirteen cervical articular process joint models were printed using several materials, printers, and printing technologies. Ultrasound video clips with the models immersed in water were recorded. Two board certified veterinary radiologists and three veterinary radiology residents reviewed the videos and responded to a survey assessing and comparing the ultrasonographic characteristics of the 3D printed models to those of the gold standard.

**Results:**

Six 3D printed models had ultrasonographic characteristics similar to the gold standard. These six models were (material, printer, printing technology): nylon PA 12, EOS Formiga P100, selective laser sintering (P = 0.99); Onyx nylon with chopped carbon fiber, Markforged Onyx Two, fused deposition modeling (P = 0.48); polycarbonate, Ultimaker 3, fused deposition modeling (P = 0.28); gypsum, ProJet CJP 660 Pro, ColorJet Printing (P = 0.28); polylactic acid, Prusa I3, fused deposition modeling (P = 0.23); and high temperature V1 resin, Form 2, stereolithography (P = 0.22).

**Conclusion:**

When assessed in water, it is possible to replicate the qualitative ultrasonographic characteristics of bone using three dimensional printed models made by combining different materials, printing technologies, and printers. However, not all models share similar qualitative ultrasonographic characteristics with bone. We suggest that the aforementioned six models be used as proxy for simulating bones or joints for use with ultrasound. In order to replicate the resistance and acoustic window provided by soft tissues, further work testing the ability of these models to withstand embedding in material such as ballistic gelatin is required.

## Introduction

### Osteoarthritis—Pathophysiology and treatment

In the equine racehorse industry, reduced athletic performance due to joint injury and lameness has been extensively reviewed [1–3]. Relevant breeding and racing statistics data as well as veterinarian reasons for losses in training in the Thoroughbred industry revealed that 53% of horses included experienced lameness and 20% of them had a significant lameness that prevented further competition following injury [4]. Osteoarthritis is a frequent cause of poor performance in horses and has been estimated to represent more than 50% of all lameness problems [5,6].

Osteoarthritis is defined as a group of diseases with a multifactorial etiopathogenesis but a similar biologic, morphologic, and clinical outcome [7–9]. Similar to people [7,9], injury to the articular cartilage, synovial membrane, subchondral bone, ligaments, or fibrous joint capsule can lead to osteoarthritis in horses [1]. Regardless of the initiating cause, an increased production of enzymes (matrix metalloproteinases, aggrecanases), inflammatory mediators (prostaglandins), free radicals, cytokines, interleukin-1, and necrosis factor-α decreases the synthesis of matrix components with enzymatic degradation of proteoglycans and collagen [1,3]. Those complex physiological reactions lead to an imbalance between synthesis and degradation of the articular cartilage with subsequent fibrillation, ulceration, cartilage loss, and subchondral bone sclerosis [1,7,9].

Glucocorticoids (GC) or other disease modifying drugs are commonly used to treat osteoarthritis. When injected intra-articular, GC act directly on nuclear steroid receptors and interrupt the inflammatory and immune cascade at several levels, thereby reducing the accumulation of inflammatory cells, enzymes, and the secretion of inflammatory mediators [2,9].

### Intra-articular injections in equine medicine

Several ultrasound guided techniques for intra-articular injections are reported. Injection techniques of the cervical articular process joints [10,11], coxofemoral joint [12,13], and medial femorotibial joint [14] have been described. Currently, the most common practical approach to learning equine intrasynovial injections involves the use of cadavers. Dye or radiographic contrast is injected into synovial structures and subsequent dissection or radiographic evaluation confirms the site of injection [12,13,15,16]. Although cadavers are anatomically the closest to live animals, their use has several disadvantages. A limited number of attempts is possible for each joint, the need for dissection results in delayed feedback, and this training method is expensive as it requires preparation, instructor availability, facility use, cadaver acquisition, and ethical considerations of animal use [17].

### Simulation based medical education (SBME)

Considering the increasing demand for training, limited patients, and focus on patient safety, ethical alternatives such as SBME are used in healthcare education to facilitate learning of medical skills [18–21]. SBME uses artificial representations to replicate clinical scenarios, promote education through experiential learning, and improve patient safety [22]. A Best Evidence Medical Education systematic review describes several important features and aspects of simulators leading to effective learning [23]. Many of those features such as provision of direct feedback, possibility to practice skills repetitively, adaptation of the difficulty level, and implementation of clinical variation can be achieved with 3D printed models [23]. In the medical field, studies have been conducted to assess the clinical applications of 3D printed models. A study discussed the creation of prosected human cadavers and other anatomical specimens to obviate the societal controversy associated with the dissection of cadaveric material in professional medical training [24]. A different study assessed the potential of 3D printing to assist in preoperative planning, help develop intraoperative guidance tools, teach patients and surgical trainees, and produce patient-specific prosthetics [25]. These examples illustrate the emerging applications of 3D printing in the medical industry.

### 3D printed models of bone and ultrasonographic assessment of 3D printed models

To date, several 3D printed models simulating bone have been described and tested for their physical and mechanical properties [21,26,27]. Limited research has been applied to the study of ultrasonographic characteristics of hard tissue substitutes. One review of tissue substitutes for ultrasound imaging states that epoxy is promising as it can be mixed with other materials to achieve a variety of acoustic properties [28]. A different study examined the change in frequency-related velocity and attenuation caused by variation in mineral content and porosity of 3D printed models made of organic epoxy matrix [29]. Quantitative ultrasonographic methods were used to test a cylindrical 3D printed model made of polyvinylchloride and to replicate human phalanges [30]. Quantitative ultrasound parameters of a phantom made of a two-part epoxy compound were assessed and compared with cortical bone [31]. In each of the aforementioned studies, only a limited number of 3D printing materials were evaluated.

### Objectives

The purpose of this study was to compare the qualitative ultrasonographic characteristics of 3D printed models of equine cervical articular process joints to that of a dissected equine cervical spine (gold standard). Each model was made using a unique combination of a 3D printing material, printer, and printing technology. The current research is part of a large-scale project aiming to create a 3D printed anatomical model of an equine neck for teaching ultrasound guided injections of the cervical articular process joints, as this is a common site of osteoarthritis in horses. It was hypothesized that some models would share similar to nearly identical ultrasonographic characteristics to the gold standard, making them an adequate training tool for ultrasound guided procedures involving bones and joints.

## Materials and methods

**Fig 1:**
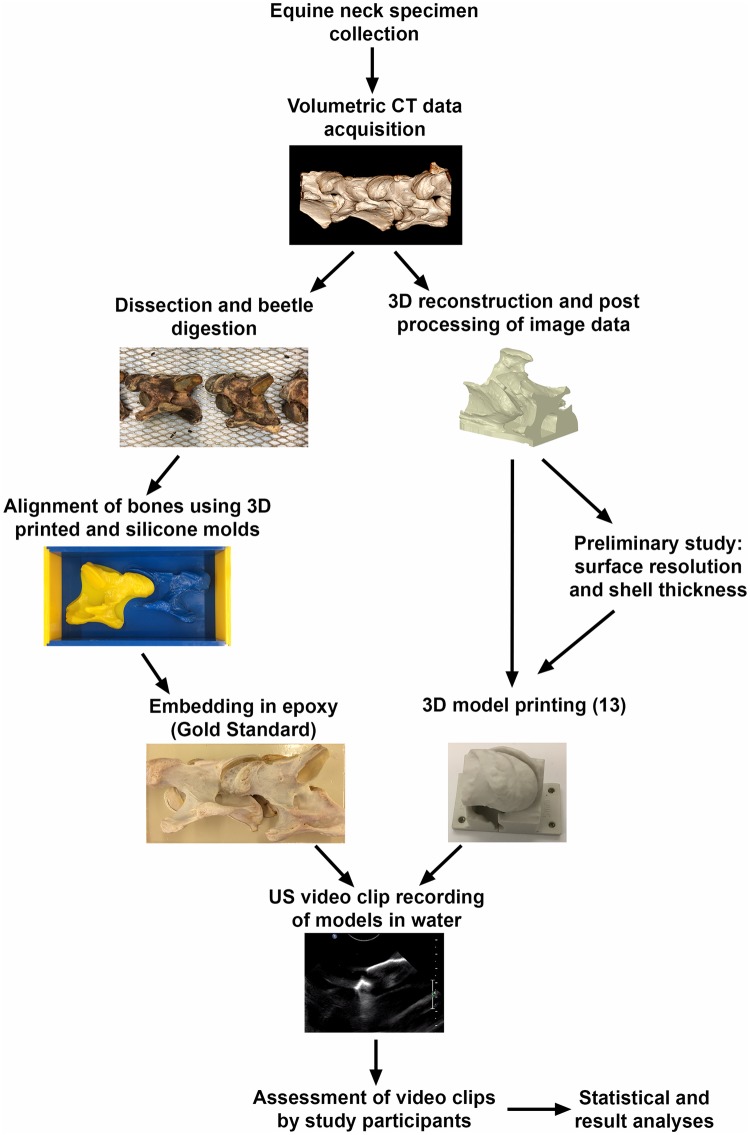
Summary of the materials and methods for the ultrasonographic evaluation of 3D printed models.

A summary of the study materials and methods is provided in Fig 1.

### Anatomical dissection

**Fig 2:**
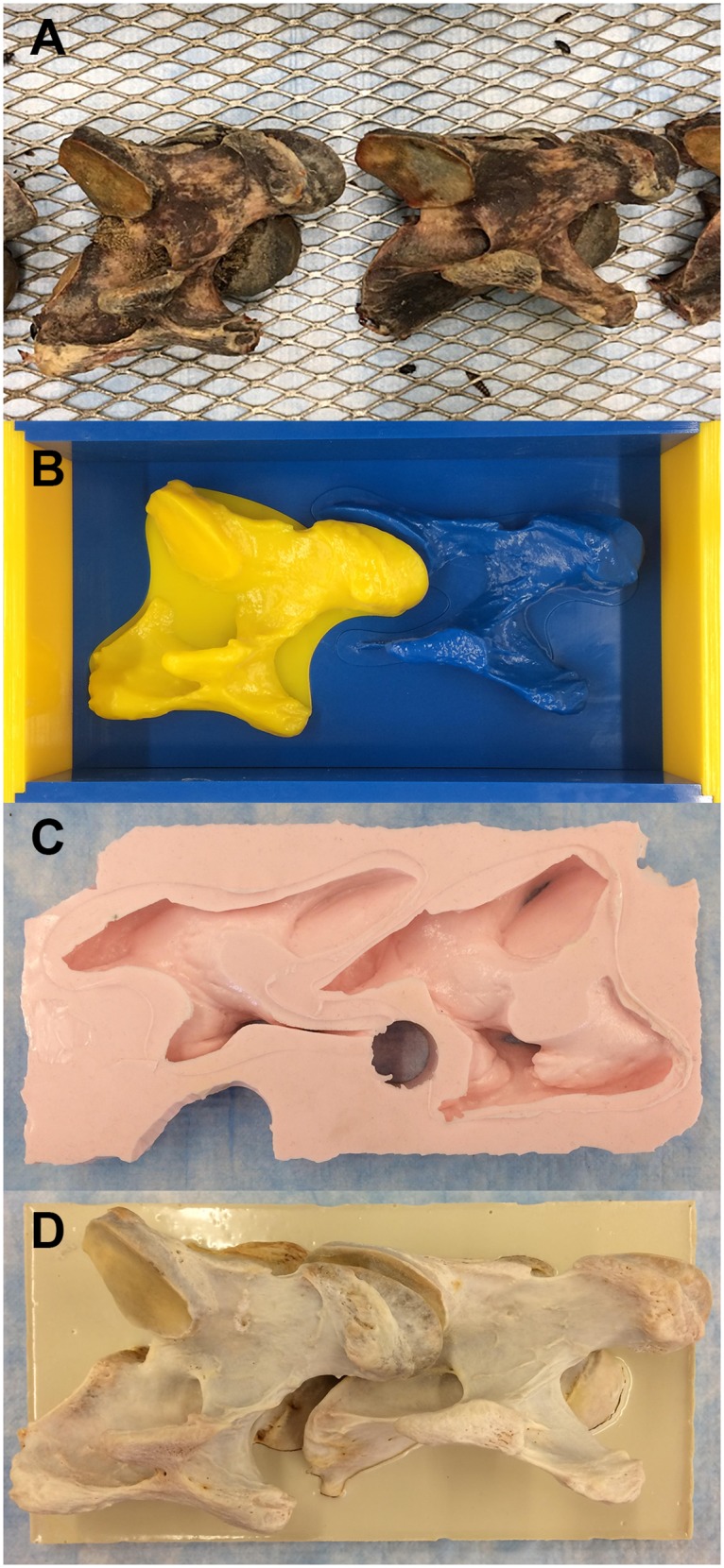
Gold standard model preparation. (A) Cervical vertebrae placed in a tray following natural dissection by beetle digestion. (B) 3D printed models of cervical vertebrae and a box, made of Vero resin. (C) Silicon mold obtained following the addition of Mold Max 20 to the 3D printed box shown in B. (D) Dissected vertebrae were inserted into the silicon mold in C, and fixated in anatomical alignment with epoxy.

The neck of a horse was obtained immediately following humane euthanasia for a reason unrelated to the current study and cut in a sagittal plane with a band saw. Vertebrae were grossly dissected and placed in a box with beetles (*Dermestes maculatus)* that fed on the soft tissues for a duration of two weeks. Following this initial period, the vertebrae were further cleared of soft tissues and placed back in the box for an additional 5 weeks (Fig 2A). At the end of the seven-week period, the specimen was removed from the box and degreased for four days with a handmade degreaser using non-dilute trichloroethylene (Univar, IL, USA). The vertebrae were bleached (hydrogen peroxide, 2.5–3%) for five days. Following bleaching, they were rinsed for two hours and allowed to air dry.

### Image acquisition

Computed tomographic images of the equine cadaver neck were obtained at the Ontario Veterinary College (Guelph, Canada). Images of the neck were acquired prior to soft tissue dissection to preserve the anatomic alignment of the cervical vertebrae and joint space width. A 16-slice detector CT scanner (GE Brightspeed CT scanner, GE Healthcare, Milwaukee, Wisconsin, United States) was used and raw data (0.625 mm slice thickness) was acquired with a standardized protocol in helical mode, 1.0-second rotation time, 0.562:1 pitch, 120 kV and 200 mAs.

### Post processing of imaging data

The images were outputted in Digital Imaging and Communications in Medicine (DICOM) format and imported into Materialise Mimics (version 19, Materialise NV, Leuvan, Belgium), a 3-D medical image processing software. Using the Mimics software, the DICOM images were transformed from 2D CT scans into 3D models. The bone CT threshold of 226 to 2599 Hounsfield units (HU) was used in Mimics. The selected bone CT threshold encompasses the wide range of bone densities, varying from 100 to 300 from trabecular bone to 2224 to 3000 HU for teeth and enamel [32]. The 3D reconstructed CT models of the vertebrae were then exported to Materialise 3-matic (version 11, Materialise NV, Leuvan, Belgium) as stereolithography (STL) files.

### Gold standard preparation

A reconstructed 3D STL model of two partial adjacent vertebrae (Fig 2B) was printed using the Stratasys J750 PolyJet printer and Vero resin. Both 3D printed vertebrae were fused together to ensure accurate positioning of the articular processes in anatomical alignment, based on the CT scan. A silicon mold was made of the fused 3D printed model using Mold Max 20 silicone (Smooth-On, Macungie, Pennsylvania, United States) (Fig 2C). The dissected vertebrae were then inserted into the mold and fixated with EpoxAcast 670 HT epoxy casting compound (Smooth-On, Macungie, Pennsylvania, United States) (Fig 2D).

### Preliminary study

Using the volumetric data acquired, 3D hollow models of varying surface resolution (50 μm, 100 μm and 200 μm) and shell thickness (1, 2, 3, 4 and 5 mm) were made with polylactic acid (printer: Ultimaker 3, printing technology: fused deposition modeling) and high temperature resin (printer: Form 2 and printing technology: SLA). Each model was manually held under water and scanned using a 5–8 MHz curvilinear probe (Philips UI22, Philips, Bothell, USA). All images were obtained in a transverse plane along the joint space, from the cranial to caudal aspect of the joint space. The ultrasonographic images were assessed and compared to the gold standard by a board certified veterinary radiologist (AZ) and veterinary radiology resident (AB). The effect of surface resolution and shell thickness on the following imaging characteristics were subjectively assessed: superficial wall (cortex) echogenicity, thickness and texture, creation of reverberation artifacts, joint space visibility, and margins. Following this initial assessment, the hollow portion of the models was filled with high temperature castable epoxy (EpoxAcast 670 HT) and the ultrasonographic assessment was repeated. Epoxy filling was tested to assess the ultrasonographic characteristics of models with a filled center, while limiting the quantity of 3D printing material used and therefore reducing the production cost.

### Printing of 3D models

**Fig 3:**
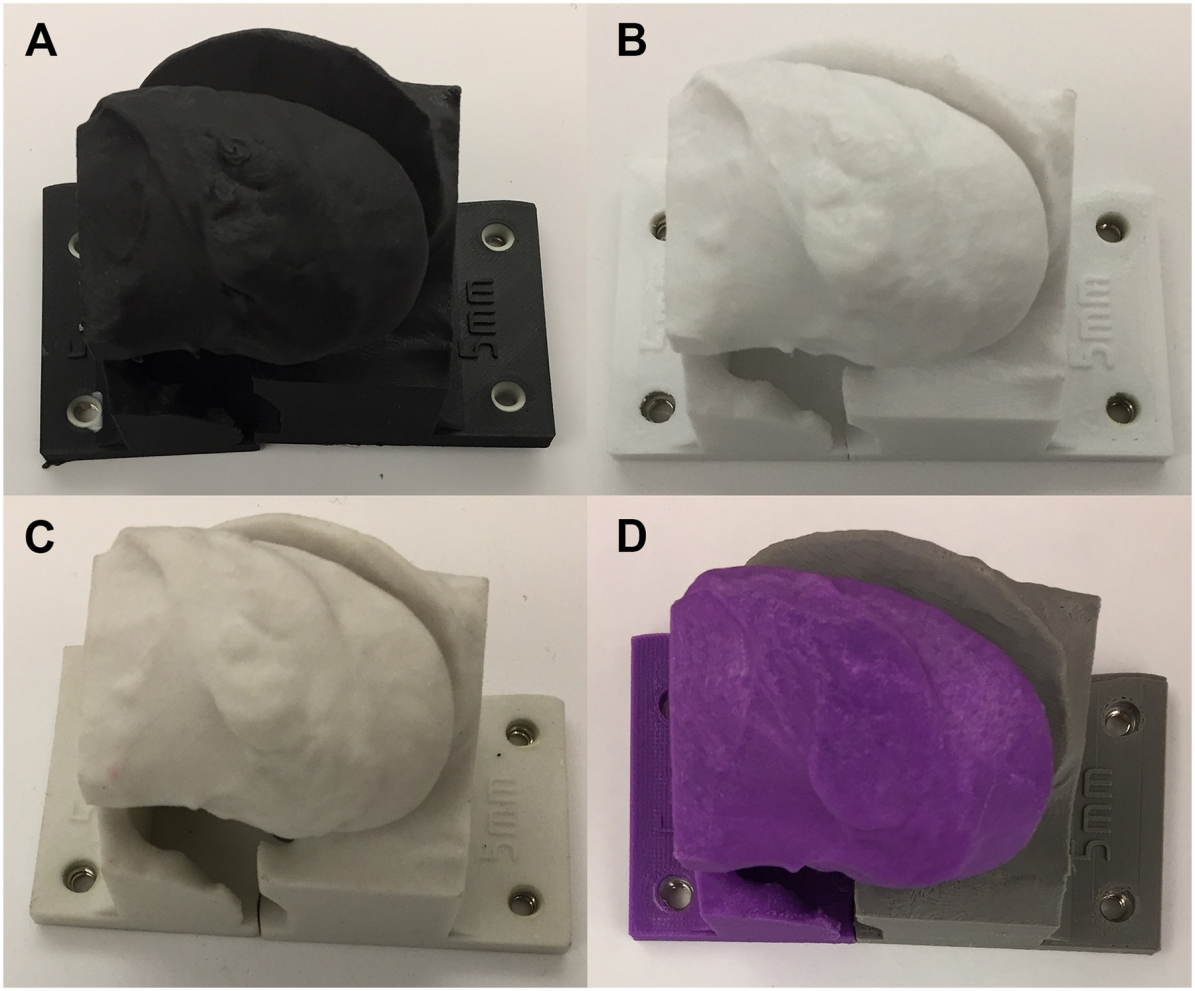
Four representative 3D printed models of an equine articular process joint. The models are printed with a surface resolution of 100 μm and a shell thickness of 5 mm. (A) Model H: Onyx nylon with chopped carbon fiber, Markforged Onyx Two, fused deposition modeling. (B) Model I: polylactic acid, Ultimaker 3 Extended, fused deposition modeling. (C) Model D: gypsum, ProJet CJP 660 Pro, ColorJet Printing. (D) Model M: polylactic acid, Makerbot Replicator +, fused deposition modeling.

Using the parameters selected from the preliminary study, a randomized complete block design study was conducted in which a total of thirteen cervical articular process joint models were printed. Each model was made using a unique combination of a 3D printing material, printer, and printing technology (Table 1 and Fig 3). Further information regarding the preprocessing steps, processing parameters and postprocessing steps are provided in S1 Table. The cost of each model was determined by each commercial company taking into account material and labor costs as well as 3D printer wear (Table 1). Models A, B and C were purchased from the company Sculpteo (Villejuif, France). Models E, F, I, J were provided by the company Shop 3D (Ontario, Canada). Models D, G, H, K, M were obtained from the company Objex unlimited Inc (Ontario, Canada). Finally, model L was made using a 3D printer retail kit owned by a third-party provider and model cost was based solely on material cost.

**Table 1:** Summary of printed models tested with their respective manufacturing information.

Models	Company	3D printer	Printing technology	Material	Production cost per model ($CAD)
A	Stratasys	Stratasys J750	PolyJet	Resin, VeroPureWhite, glossy finish	297
B	Stratasys	Stratasys J750	PolyJet	Resin, VeroPureWhite, matte finish	297
C	EOS	EOS Formiga P100	SLS^a^	Nylon PA 12	127
D	3D Systems	ProJet CJP 660 Pro	CJP^b^	Gypsum	120
E	Ultimaker	Ultimaker 3 Extended	FDM^c^	PLA^d^	40
F	Formlabs	Form 2	SLA^e^	Resin, high temperature V1	100
G	3D Systems	ProJet 6000HD	SLA	Resin, Visijet SL Black	200
H	Markforged	Markforged Onyx Two	FDM	Onyx, nylon with chopped carbon fiber	80
I	Ultimaker	Ultimaker 3 Extended	FDM	Polycarbonate	60
J	Formlabs	Form 2	SLA	Resin, Grey V3	80
K	3D Systems	ProJet 5500X	MJP^f^	Resin, Visijet CE-BK	250
L	Prusa	I3 3D printer retail kit	FDM	PLA	10
M	Makerbot	Makerbot Replicator +	FDM	PLA	40
N	Positive control (dissected specimen)	N/A^g^	N/A	N/A	N/A

^a^ Selective Laser Sintering

^b^ ColorJet Printing

^c^ fused deposition modeling

^d^ Polylactic acid

^e^ Stereolithography

^f^ MultiJet Printing

^g^ N/A = Not applicable

### Ultrasonographic assessment of 3D printed models

**Fig 4:**
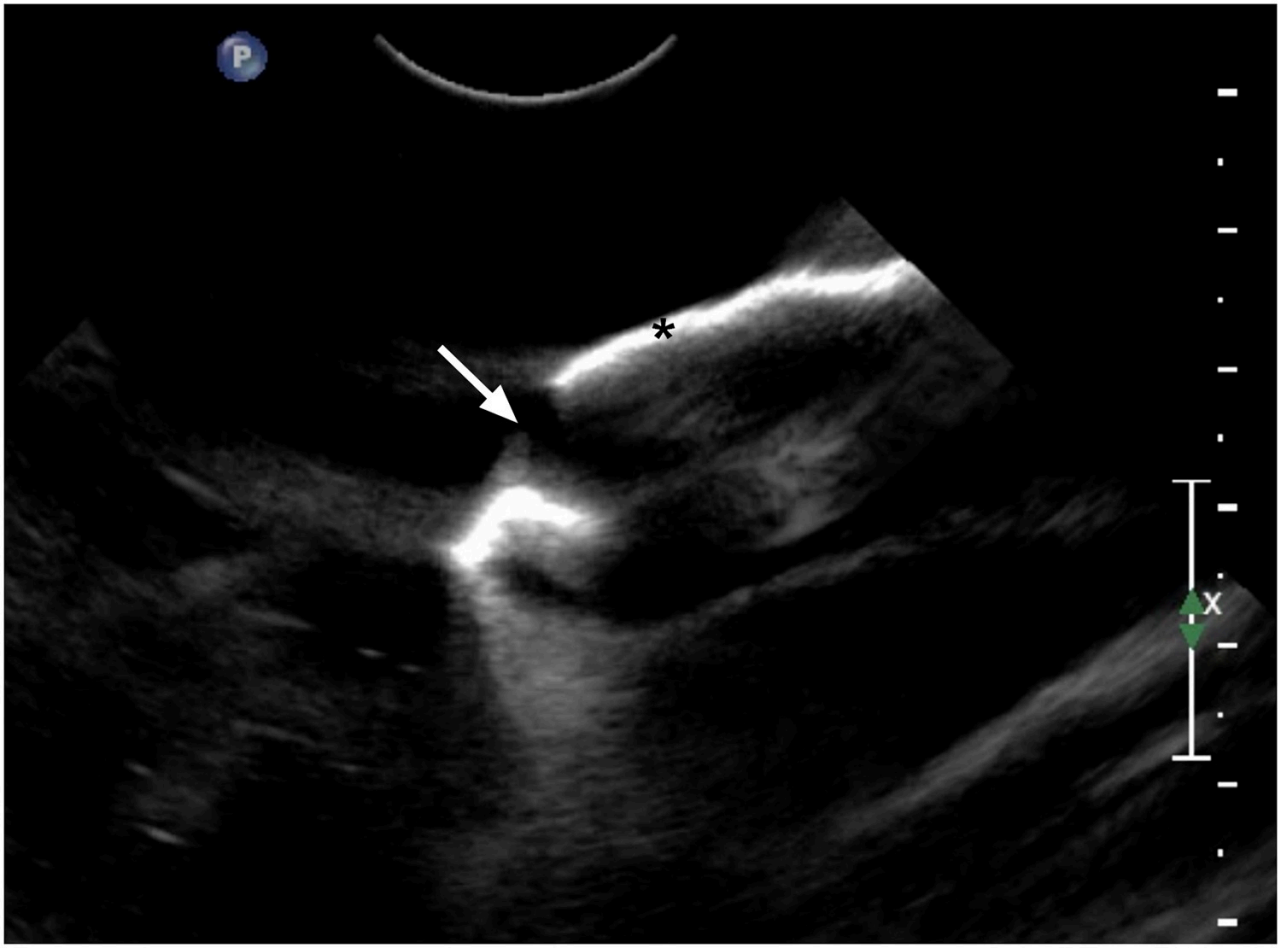
Ultrasound image of model C immersed in water. Model C made of nylon PA 12 using an EOS Formiga P100 printer and selective laser sintering printing technology. Note the characteristic hyperechoic appearance created by the strong reflection of the ultrasound beam by the model, simulating bone (*). The joint space is visible as a gap between the cranial (left) and caudal (right) articular processes (white arrow).

Each model was screwed to the bottom of a metal tin using bolts inserted through drilled holes and maintained in place with metal and rubber washers. The tins were filled with tap water to immerse the models. Using the same ultrasonographic equipment and technique described above, a video clip of each printed model immersed in water was recorded (Fig 4). Each of the thirteen 3D printed models was attributed a letter from “A” to “M”. Two ultrasound video clips of the bone specimen (gold standard) were also recorded. One of the two videoclips of the bone specimen was labelled “gold standard”. The second video clip was attributed a letter (“N”) similar to the 3D printed models and was used as a positive control.

All videos clips were adjusted for time gain compensation (Iscan function) followed by adjustment of the overall gain to optimize image quality. A single focal point was used and adjusted along the beam axis to maximize lateral image resolution. The depth of the field of view was standardized for all models by adjusting the scale on the ultrasonographic display. The longest uninterrupted video clip duration achievable on the ultrasound machine was selected (10 seconds). Each video clip was exported in DICOM format into a commercially available DICOM image viewing software (Horos, 64-bit, version 3.2.1, Nimble CO LLC d/b/a Purview, USA) and converted into MOVIE format.

Five participants including two veterinary radiologists and three veterinary radiology residents with varying experience (1–10 years) were recruited. Participants were recruited in accordance with research ethics guidelines and the study was approved by the research ethics board (REB) at the University of Guelph, Canada (REB# 612–490). Each participant was provided with online access to the recorded video clips. Participants were asked to compare each video clip (from A to N) to the gold standard in a predetermined randomized order. For each model, a six-question survey was answered. The survey aimed to assess the similarity or dissimilarity between ultrasonographic characteristics of the models and the gold standard (Table 2). The answers were digitally recorded on a 10-point Likert-like scale by drawing a vertical line according to the degree of agreement (from strongly disagree to strongly agree) (S1 File). All surveys were anonymously returned using a third party and data was tabulated in a Microsoft Excel document.

**Table 2:** Survey statements comparing the ultrasonographic characteristics of each model to the gold standard. For each model, participants recorded their level of agreement with the proposed statements on a 10 point modified Likert-like scale. Similarity or dissimilarity between the ultrasonographic characteristics of the models and the gold standard was based on the recorded level of agreement.

Questions	Description
1	The ultrasonographic features of the superficial wall (cortex) of the model are comparable to the gold standard.
2	The reverberation artifact at the surface of the model is comparable to the gold standard.
3	The articular process joint space size and visibility of the model is comparable to the gold standard.
4	Internal structures/echoes are noted in the model, unlike the gold standard.
5	Artifacts are produced by the model that would prevent its use as a training model compared to the gold standard.
6	The model would be an acceptable replacement to the gold standard as a training tool for ultrasound guided procedures involving bones/joints.

Statistical analyses were selected and performed by a statistician (GM). The survey scores of each model (A to M) were compared to the survey scores of the positive control (model N) for each participant. Individual question scores as well as summed scores for all six questions were analyzed for mean/median differences of the models compared to the positive control. For the summed question scores, data was converted to absolute values. A mixed model ANOVA having participant identification as a random effect and model as a fixed effect was applied. Residuals were checked for normality with a Shapiro Wilk test. Friedmans ANOVA was used if the data did not meet the assumptions of normality, even after a log transform was applied. Post hoc tests were based on a Dunnetts with significance set at p≤ 0.05. A trend towards significance was interpreted as a p value between 0.05 and 0.1. The null hypothesis consisted of no significant difference between the qualitative ultrasonographic characteristics of the models and the gold standard.

## Results

### Preliminary study

**Fig 5:**
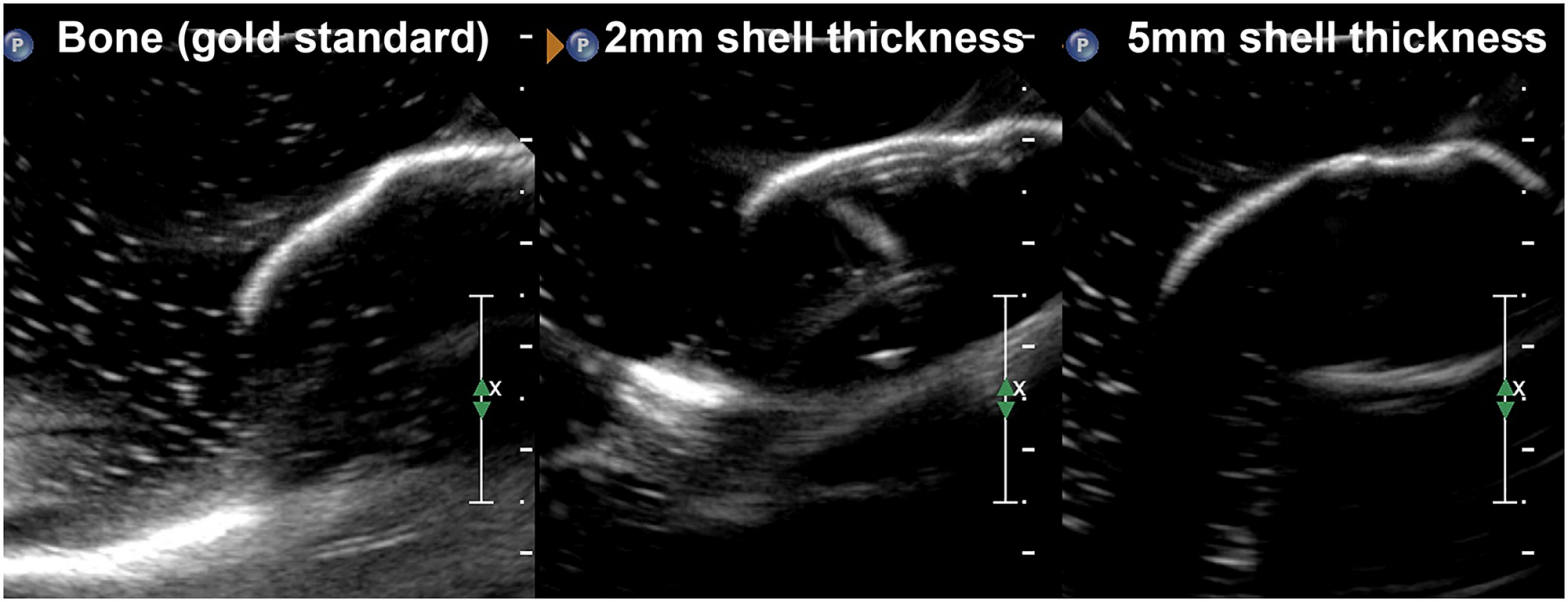
Ultrasound images comparing two 3D printed models to the gold standard. Both models are printed using the SLA printing technology and a surface resolution of 100 μm. The shell thickness of both models differs (2 mm vs. 5 mm). Note the similar cortical surface of bone (gold standard) and the 5 mm thick model, whereas multiple reflections are displayed beneath the surface of the 2 mm thick model.

The surface resolutions assessed did not affect the qualitative ultrasonographic characteristics of the superficial wall (cortex) of the models. It also did not create a reverberation artifact, or modify the joint space visibility and margins. A surface resolution of 100 μm was selected as most of the 3D printing technologies used in the current study could print at 100 μm; the one exception being the PolyJet printing technology printing at 27 μm only. Also, a higher surface resolution (e.g. 50 μm) is associated with a longer production time and higher production cost. A shell thickness of 5 mm produced ultrasonographic images with the most realistic cortex (Fig 5). Unlike the gold standard, thicknesses varying from 1 to 4 mm did not completely reflect the echoes, creating a reverberation artifact seen as multiple reflections displayed beneath the real reflector (Fig 5). A thickness of 1 mm was also impractical due to fragility and friability. The addition of high temperature castable epoxy (EpoxAcast 670 HT) in the hollow portion of the models created a hyperechoic interface under the model surface, which was not observed with the gold standard. Therefore, the use of the filling material was discontinued for the remainder of the study.

### Ultrasonographic assessment of 3D printed models

**Fig 6:**
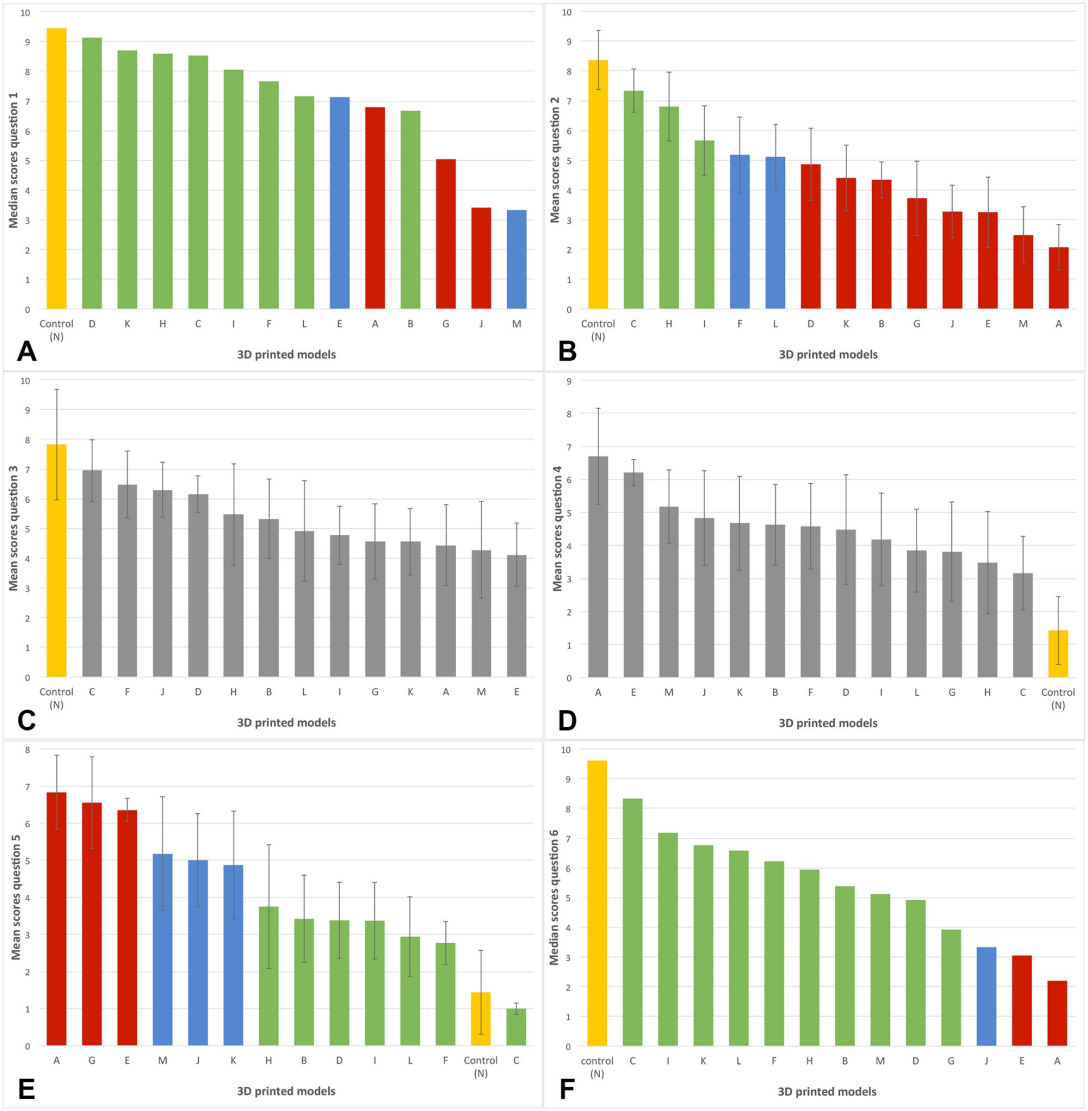
Mean score with standard error for questions 2–5 (graph B-E) and median score for questions 1 and 6 (graph A^a^ and F)^b^. Yellow: positive control, green: models not statistically different from the positive control, blue: models trending towards a statistical difference when compared to the positive control, red: models statistically different from the positive control. Question 3 and 4 (graphs C and D respectively) did not reach a significant F value and post-hoc tests were therefore not performed; all models are displayed in grey. ^a^ Note that the model rank order based on the p values and medians differs for graph A since the Friedmans test from which the p values were calculated is based on the rank sums and not the medians. ^b^ The mean and median scores are respectively reported for normally and non-normally distributed data.

**Fig 7:**
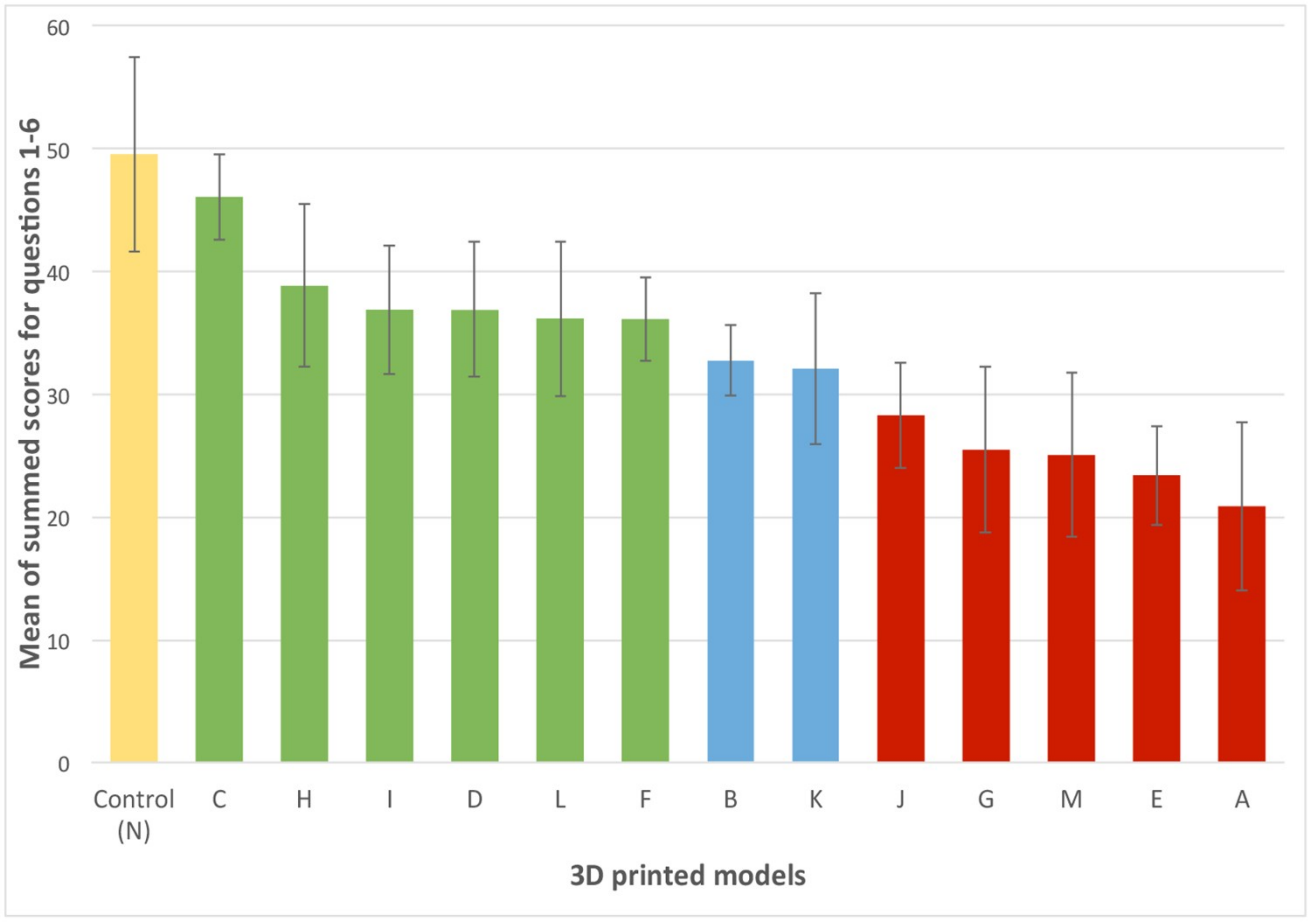
Mean score with standard error for the summed question scores with standard error. Yellow: positive control, green: models not statistically different from the positive control, blue: models trending towards a statistical difference when compared to the positive, red: models statistically different from the positive control.

Using the tabulated data gathered from each participant for each question (S2 File), the score of individual questions and the sum of question scores were calculated and are reported in Figs 6 and 7, respectively. Model ranking based on p values for individual questions and sum of question scores is reported in Table 3. Data was normally distributed for question 3, 4, 5 and for the summed question scores. Data was non-normally distributed for question 1 and 6. Question 3 and 4 did not reach a significant F value and post-hoc tests were not performed.

**Table 3:** Model rankings based on the p values for individual questions and sum of question scores.

Rank^a^	Question 1	Question 2	Question 5	Question 6	Sum of question scores
1	D^b^ (1.00)^c^	C (0.98)	C (1.00)	C (0.99)	C (0.99)
2	C (0.99)	H (0.79)	F (0.95)	I (0.99)	H (0.48)
3	H (0.99)	I (0.18)	L (0.90)	L (0.99)	I (0.28)
4	K (0.98)	F (0.075)	I (0.69)	H (0.91)	D (0.28)
5	L (0.93)	L (0.06)	D (0.68)	K (0.80)	L (0.23)
6	I (0.74)	D (0.04)	B (0.66)	F (0.63)	F (0.22)
7	F (0.27)	K (0.013)	H (0.47)	D (0.45)	B (0.07)
8	B (0.17)	B (0.012)	K (0.09)	B (0.43)	K (0.05)
9	M (0.07)	G (0.002)	J (0.07)	M (0.25)	J (0.01)
10	E (0.07)	E (0.0007)	M (0.05)	G (0.11)	G (0.003)
11	A (0.03)	J (0.0007)	E (0.005)	J (0.06)	M (0.002)
12	J (0.03)	A (<0.0001)	G (0.003)	A (0.02)	E (0.0008)
13	G (0.02)	M (<0.0001)	A (0.001)	E (0.02)	A (0.0002)

^a^ A rank of 1 indicates the most similarity

^b^ Model letter

^c^ p value

A broad variation in the ultrasonographic characteristics of the models was observed. When questions were considered individually, model D had the cortex with the most similar appearance to the gold standard (p = 1.00) (Fig 6A). Seven other models (C, H, K, L, I, F, B,) had a cortex with ultrasonographic characteristics that did not significantly differ from the positive control. Models M and E trended towards a significant p value. The reverberation artifact noted at the surface of the gold standard was most accurately depicted by model C (p = 0.98) (Fig 6B). No significant difference regarding the reverberation artifact was found for models H and I and near significance was obtained for model F and L. Model C produced the least artifacts (p = 1.00), followed by models F, L, I, D, B and H (Fig 6E). Models K, J and M had p values trending towards a significant difference when compared to the positive control. Model C was determined to be the most acceptable replacement to the gold standard as a training tool for ultrasound guided procedures involving bone and joints (p = 0.99) along with nine other models (I, L, H, K, F, D, B, M, G) (Fig 6F). Model J trended towards a significant p value.

When all question scores were summed, six models had ultrasonographic characteristics that were not statistically different from the positive control (p> 0.1). These included models C, H, I, D, L and F (Fig 7). Two models (B and K) were not different from the positive control but approached the significance level.

## Discussion

### 3D printed models

Simulation based medical education uses artificial representations to replicate clinical scenarios and promote education through experiential learning. Described features and best practices of simulation based medical education emphasize the importance of simulation fidelity, which guided the purpose of the present study. This work supported our hypothesis that a variety of 3D printed models share similar ultrasonographic characteristics with a dissected equine cervical spine, making them an acceptable training tool for ultrasound guided procedures involving bones and joints.

The p value adjustment due to the multiple comparisons was conservative. To minimize the possible bias induced by the conservative p value adjustment, falsely decreasing the statistical significance of some models, only models non-statistically different from the gold standard were considered a good replacement for bone in the creation of 3D printed models. This ensured the exclusion of models categorized as trending towards a statistical significance with the conservative adjustment that would, in fact, be considered as statistically different without the conservative adjustment.

Model C (nylon PA 12, EOS Formiga P100, SLS) was found to be the most realistic replacement of bone. Other models including model H (onyx nylon with chopped carbon fiber, Markforged Onyx Two, FDM), model I (polycarbonate, Ultimaker 3 Extended, FDM), model D (gypsum, ProJet CJP 660 Pro, CJP), model L (PLA, I1 3D printer retail kit, FDM), and model F (high temperature V1 resin, Form 2, SLA) were also considered acceptable. The finding of high temperature V1 resin (model F) as a good replacement of bone agrees with a previous study [28] stating that epoxies are a good hard tissue substitute. Model L was not statistically different from the gold standard and therefore is considered an appropriate replacement of bone in simulation testing. However, this model was friable and showed occasional discontinuities in the model surface, especially along the margins of the articular processes. Although the assessment of the model mechanical properties and resistance was not the aim of the current study, the poor quality of model L is not favorable in the creation of resistant and long-lasting 3D printed models designed for repetitive use.

Models with shell thicknesses ranging from 1 mm to 5 mm were tested as part of the preliminary study. With the goal to create affordable and therefore accessible 3D printed models, production costs were considered. Shell thickness being related to both time for production and amount of material required per model, it directly impacts production cost. For a thicker model, the printing time and amount of printing material required is greater, resulting in a higher cost. As part of the shell thickness testing, we found that a thickness of 5 mm mimics the gold standard cortex the closest. Unlike the gold standard, thicknesses varying from 1 to 4 mm did not completely reflect the ultrasound beam. The impact of shell thickness on the ultrasound images obtained is a function of the interaction of ultrasound with matter. As ultrasound energy propagates through a medium, interactions include reflection, refraction, scattering, and absorption [33]. Reflection of the ultrasound beam at a boundary between two media occurs because of the differences in the acoustic impedance of the two structures. In the current study, partial reflection of the ultrasound beam occurred when it encountered the surface of the 3D printed model after traveling in water. The concept of reflection is important to understand the reverberation artifact. Reverberation artifact consists of multiple reflections occurring between two strong reflectors, or between the transducer and a strong reflector [34]. These multiple reflections are displayed beneath the real reflector at intervals equal to the distance between the transducer and the real reflector. As shown in Fig 5, these reflections were present under the surface of the thinner models, especially models printed at a thickness of 1 mm. A clear explanation clarifying the presence of a reverberation artifact noted with the thinner shell thicknesses remains unclear. However, it would be reasonable to think that these models acted as a stronger reflector or simply allowed better visualization of the artifact under their thinner surface.

As part of the preliminary study, we tested the addition of high temperature castable epoxy (EpoxAcast 670 HT) in the hollow portion of the models. Unlike the gold standard, this created a hyperechoic interface under the model surface. Accordingly, all the 3D printed models created were made hollow without any specific internal structures. This highlights the possibility of employing 3D printed light parts with a hollow center to reproduce joints, minimizing the production cost by decreasing the amount of 3D printing material used. This also contributes to the creation of models that are easy to use, manipulate and transport due to their light weight.

According to the observations made as part of the preliminary study, standardizing the shell thickness and surface resolution of most models minimized the numbers of variables tested. This standardization allowed focusing the assessment on the ultrasonographic characteristics of printing materials, printers, and printing technologies. It would be ideal to independently assess printing materials, printers, and printing technologies by standardizing two of the three variables across all models. However, this could not be performed due to limitations inherent to 3D printing. Each individual printer can only print with one technology and one or more materials, depending on the printer. Despite this limitation, among the models tested in the current study, two were made using the same printer and printing technology but different materials. Model E and I were both printed with a Ultimaker 3 Extended printer, using the fused deposition modeling technology. Model E was made of polylactic acid and model I was made of polycarbonate. Whereas model E was statistically different from the gold standard, model I was not. Although additional similar comparisons would be required to draw appropriate conclusions, this implies that printing material alone can significantly change the ultrasonographic characteristics of 3D printed models. Also, of all the models tested, two were made of nylon (models C and H). Both of these models were not statistically different from the gold standard based on their qualitative ultrasonographic properties. We postulate that nylon may be a superior printing material to replicate bone ultrasonographically and should be considered for the creation of 3D printing models to practice ultrasound guided intra-articular injections.

As part of the study, six printing technologies were assessed. Among the models that were not statistically different from the gold standard, four printing technologies were used: selective laser sintering, ColorJet Printing, stereolithography and fused deposition modeling. This finding suggests that a sole printing technology is not superior in the creation of 3D printed models replicating bone ultrasonographically. This finding reinforces the statement that printing technology should be selected based on several criteria such as intended use, printing time, availability, cost, materials, color, biocompatibility, sterilization capability, resistance, transparency, molding, or casting properties [35]. Even though an exhaustive description of each printing technology is beyond the scope of this study, each technology is unique. Some printing technologies are more labor intensive. Stereolithography (SLA) is an example of vat photopolymerization, which involves several steps and uses a high-intensity light source, a vat of photo-curable liquid resin, and a controlling system to produce 3D printed models. Resin is solidified by a light source in successive 2D layers as a platform is progressively lowered or raised. The excess resin is removed by rinsing with a solvent, and the model is cured in a UV chamber [35]. In the current study, printing was performed in such an orientation that no supports were in contract with the model surface, avoiding alteration in the parameters investigated ultrasonographically. ColorJet Printing (CJP) is a type of binder jetting technology and uses a print head to jet a liquid binding agent onto a bed made of fine powder. The powder bed is selectively bonded where the liquid is deposited. After each layer completion, new powder is deposited for the new layer. By gluing the particles together, the part is built up layer by layer. The unbound powder is blown off and the last step consists of infiltrating the model with cyanoacrylate wax or resin for solidification. A support structure is not needed because the model is continuously supported by unbounded powder during fabrication. Selective laser sintering (SLS) is an example of powder bed fusion which uses a high-power laser of electron beam to fuse small particles of material into a desired 3D shape layer by layer. After a layer is fused, the powder bed is lowered by one layer thickness and a new layer is applied on top. This technology does not require the use of a support as described for binder jetting [35]. Similar to CJP, SLS requires removal of unbound powder from the surface of the models using pressurized air.

While a wide variety of 3D printers are available, some are easily accessible while others are more expensive. In order to create readily accessible 3D printed models and promote learning, it is important to consider the financial investment related to the acquisition of a 3D printer. Of the models non-statistically different from the gold standard, models C and D were made using commercial printers (EOS Formiga P100 and ProJet CJP 660 Pro respectively). On the other hand, models H, I and F were made using more affordable and accessible printers (Markforged Onyx Two, Ultimaker 3 Extended, Form 2 respectively).

### Limitations

A few limitations were encountered in this study. The number of participants was limited and recruitment of additional radiologists or radiology residents could have increased statistical power. The main investigators did not participate in the assessment of the videos in order to remove any potential bias. Of more importance was the subjectivity of the criteria used to compare the videos of the models to the gold standard. Assessment of features such as articular process joint visibility and model surface/cortex could have introduced some variability due to personal interpretation. Subjectivity was limited by providing a document of descriptions and examples to guide participants in their assessment (S3 File). Videos were also recorded and standardized by the investigators to avoid operator variability. As briefly mentioned above, there are inherent limitations in 3D printing. Although the printing resolution was standardized for most models, the Polyjet printing technology used for models A and B could only achieve a higher resolution of 27μm.

## Conclusions

This study provides knowledge for further educational training involving the printing of 3D models simulating bone for ultrasound guided procedures. According to their qualitative ultrasonographic properties, the following models are a good replacement of bone: nylon PA 12 (EOS Formiga P100, SLS); Onyx nylon with chopped carbon fiber (Markforged Onyx Two, FDM); polycarbonate (Ultimaker 3, FDM); gypsum (ProJet CJP 660 Pro, CJP); polylactic acid (Prusa I3, FDM), and high temperature V1 resin (Form 2, SLA). Conditional to their ability to withstand ballistics gel embedding, these models could be used for the creation of a larger 3D printed anatomical model, such as a complete equine neck for teaching ultrasound guided injections of the cervical articular process joints.

## Supporting information

S1 Table Preprocessing, processing and postprocessing information for each three-dimensional printed models. (DOCX)

S1 File Survey assessing the three-dimensional printed models ultrasonographic features as compared to the gold standard. (DOCX)

S2 File Tabulated Likert-like scale scores. (XLSX)

S3 File Document of descriptions and examples to guide and help participants in their video assessment of the models. (PDF)

## References

[1] de Souza MV. Osteoarthritis in horses–Part 1: relationship between clinical and radiographic examination for the diagnosis. Braz. Arch. Biol. Technol. 2016; 59.

[2] Goodrich LR, Nixon AJ. Medical treatment of osteoarthritis in the horse–A review. Vet J 2006;171(1): 51–69. 10.1016/j.tvjl.2004.07.008 16427582

[3] McIIwraith CW, Frisbie DD, Kawcak CE. The horse as a model of naturally occurring osteoarthritis. Bone Joint Res. 2012;1(11): 297–309. 10.1302/2046-3758.111.2000132 23610661 PMC3626203

[4] Jeffcott LB, Rossdale PD, Freestone J, Frank CJ, Towers-Clark PF. An assessment of waistage in Thoroughbred racing from conception to 4 years of age. Equine Vet J. 1982;14(3): 185–198. 7106081 10.1111/j.2042-3306.1982.tb02389.x

[5] Kidd JA, Fuller C, Barr ARS. Osteoarthritis in the horse. Equine Vet Educ. 2001;13(3):160–168.

[6] De Lasalle J, Alexander K, Olive J, Laverty S. Comparisons among radiography, ultrasonography and computed tomography for ex vivo characterization of stifle osteoarthritis in the horse. Vet Radiol Ultrasound. 2016;57(5):489–501. 10.1111/vru.12370 27237699

[7] Brandt KD, Dieppe P, Radin E. Etiopathogenesis of osteoarthritis. Med Clin North Am. 2009; 93(1): 1–24, xv 10.1016/j.mcna.2008.08.009 19059018

[8] Radin EL, Burr BD, Caterson B, Fyhrie D, Brown TD, Boyd RD. Mechanical determinants of osteoarthrosis. Semin Arthritis Rheum. 1991; 21(3 Suppl 2):12–21. 1796301 10.1016/0049-0172(91)90036-y

[9] Egeman A, Kesmezacar H, Akgun I. Intraarticular injections (corticosteroid, hyaluronic acid, platelet rich plasma) for the knee osteoarthritis. World J Orthop. 2014;5(3): 351–361. 10.5312/wjo.v5.i3.351 25035839 PMC4095029

[10] Mattoon JS, Drost WT, Grguric MR, Auld DM, Reed SM. Technique for equine cervical articular process joint injection. Vet Radiol Ultrasound. 2004;45(3): 238–240. 15200263 10.1111/j.1740-8261.2004.04042.x

[11] Purefoy Johson J, Stack JD, Rowan C, Handel I, O’Leary JM. Ultrasound-guided approach to the cervical articular process joints in horses; a validation of the technique in cadavers. Vet Comp Orthop Traumatol. 2017;30: 165–171. 10.3415/VCOT-16-09-0139 28094412

[12] David F, Rougier M, Alexander K, Morisset S. Ultrasound-guided coxofemoral arthrocentesis in horses. Equine Vet J. 2007;39(1): 79–83. 17228601 10.2746/042516407x153093

[13] Whitcomb MB, Vaughan B, Katzman S, Hersman J. Ultrasound‐guided injections in horses with cranioventral distension of the coxofemoral joint capsule: feasibility for a cranioventral approach. Vet Radiol Ultrasound. 2016;57: 199–206. 10.1111/vru.12323 26748616

[14] Kleider N. How to Inject the Medial Femorotibial Joint Recess Under Ultrasound Guidance. AAEP proceedings. 2013;59: 220–225.

[15] Herdrich MRA, Arrieta SE, Nelson BB, Frisbie DD, Morrman VJ. A technique of needle redirection at a single craniolateral site for injection of three compartments of the equine stifle joint. Am J Vet Res. 2017;78: 1077–1084. 10.2460/ajvr.78.9.1077 28836846

[16] Nielsen JV, Berg LC, Thoefner MB, Thomsen PD. Accuracy of ultraosound-guided intra-articular injection of cervical fact joints in horses; a cadaveric study. Equine Vet J. 2003;35(7): 637–661. 14649356 10.2746/042516403775696366

[17] Fox V, Sinclair C, Bolt DM, Lowe J, Weller R. Design and validation of a simulator for equine joint injections. J Vet Med Educ. 2013;40(2): 152–157. 10.3138/jvme.0912-083R1 23709111

[18] Ziv A, Wolpe PR, Small SD, Glick S. Simulation-Based Medical Education: An Ethical Imperative. Acad Med. 2003;78(8): 783–788. 12915366 10.1097/00001888-200308000-00006

[19] Sorensen JL, Ostergaard D, LeBlanc V, Ottesen B, Konge B, Dieckmann P et al Design of simulation-based medical education and advantages and disadvantages of in situ simulation versus off-site simulation. BMC Med Educ. 2017;17(1): 20 10.1186/s12909-016-0838-3 28109296 PMC5251301

[20] Motola I, Devine LA, Chung HS, Sullivan JE, Issenberg SB. Simulation in healthcare education: A best evidence practical guide. Med Teach. 2013;35(10): 1511–1530. 10.3109/0142159X.2013.818632 23941678

[21] Favier V, Zemiti N, Caravaca Mora O, Subsol G, Captier G, Lebrun R et al Geometric and mechanical evaluation of 3D-printing materials for skull base anatomical education and endoscopic surgery simulation—A first step to create reliable customized simulators. PLoS ONE. 2017;12(12). 10.1371/journal.pone.0189486 29252993 PMC5734742

[22] Al-Elq AH. Simulation-based medical teaching and learning. J Family Community Med. 2010;17(1): 35–40. 10.4103/1319-1683.68787 22022669 PMC3195067

[23] Issenberg SB, McGahie WC, Petrusa ER, Gordon DL, Scalese RJ. Features and uses of high-fidelity medical simulations that lead to effective learning: a BEME systematic review. Med Teach. 2005;27(1): 10–28. 10.1080/01421590500046924 16147767

[24] McMenamin PG, Quayle MR, McHenry CR, Adams JW. The production of anatomical teaching resources using three-dimensional (3D) printing technology. Anat Sci Educ. 2014;7(6): 479–486. 10.1002/ase.1475 24976019

[25] Chae MP, Rozen WM, McMenamin PG, Findlay MW, Spychal RT, Hunter-Smith DJ. Emerging Applications of Bedside 3 D Printing in Plastic Surgery. Front Surg. 2015;2: 25 10.3389/fsurg.2015.00025 26137465 PMC4468745

[26] Mowry SE, Jammal H, Myer C 4th, Solares CA, Weinberger P. A Novel Temporal Bone Simulation Model Using 3D Printing Techniques. Otol Neurotol. 2015; 36(9): 1562–1565. 10.1097/MAO.0000000000000848 26375979

[27] Takahashi K, Morita Y, Ohshima S, Izumi S, Kubota Y, Tamamoto Y et al Creating an Optimal 3D Printed Model for Temporal Bone Dissection Training. Ann Otol Rhinol Laryngol. 2017;126(7): 530–536. 10.1177/0003489417705395 28420248

[28] Culjat MO, Goldenberg D, Tewari P, Singh RS. A review of tissue substitutes for ultrasound imaging. Ultrasound Med Biol. 2010;36: 861–873. 10.1016/j.ultrasmedbio.2010.02.012 20510184

[29] Tatarinov A, Pontaga I, Vilks U. Modeling the influence of mineral content and porosity on ultrasound parameter in bone by using synthetic phantoms. Mech Compos Mater. 1999;35(2): 147–154.

[30] Barkmann RLS, Stampa B, Sakata S, Heller M, Gluer CC. Assessment of the geometry of human finger phalanges using quantitative ultrasound in vivo. Osteoporos Int. 2000;11(9):745–755. 10.1007/s001980070053 . 11148802

[31] Clarke AJ, Evans JA, Truscott JG, Milner R, Smith MA. A phantom for quantitative ultrasound of trabecular bone. Phys Med Biol. 1994;39: 1677–1687. 10.1088/0031-9155/39/10/011 15551538

[32] Formlabs. How to 3D Print Anatomical Models for Preoperative Planning and Enhanced Patient Consent [Analysis in brief on the Internet]. Somerville; 2017 [cited 2019 April 28]. 14 p. https://archive-media.formlabs.com/upload/Formlabs-White-Paper_How-to-3D-Print-Anatomical-Models.pdf

[33] Bushberg JT, Seibert JA, Leidholdt EM, Boone JM. The Essential Physics of Medical Imaging. 3^rd^ ed Philadelphia: Kippincott Willians & Wilkins, 2012 Chapter 14: Ultrasound; p. 501–576.

[34] Kremkau FW. Sonography Principles and Instruments. 9^th^ ed St. Louis: Elsevier, 2016 Chapter 6: Artifacts; p. 183–216.

[35] Mitsouras P, Liacouras P, Imanzadeh A, Cai T. Medical 3D Printing for the Radiology. Radiographics. 2015;35(7):1965–1988. 10.1148/rg.2015140320 26562233 PMC4671424

