# Supplementary material for: Various 3D printed materials mimic bone ultrasonographically: 3D printed models of the equine cervical articular process joints as a simulator for ultrasound guided intra-articular injections
Source: PLoS One. 2019 Aug 6;14(8):e0220332. doi: 10.1371/journal.pone.0220332 (PMC6684155; doi:10.1371/journal.pone.0220332)
Supplement: S1 Table — (DOCX) [file pone.0220332.s001.docx]

| Models | Preprocessing steps and processing parameters ^a^ | Postprocessing steps |
| --- | --- | --- |
| A | Support material 706 was utilized to adhere the model to the build platform. The model was oriented so that no support material was present in the area subsequently being probed by the ultrasound. This produced a glossy surface that was examined by the ultrasound. The layer thickness was set to 27 microns. | The support material was removed using the Stratasys Polyjet Waterjet system. |
| B | Support material 706 was applied to all surfaces. This produced a matte surface that was examined by the ultrasound. The layer thickness was set to 27 microns. | The support material was removed using the Stratasys Polyjet Waterjet system. |
| C | The layer thickness was set to 100 microns. | The part was cleaned off using a brush and an air blower. |
| D | The monochromatic print was set to the layer thickness was set to 100 microns. | The part was cleaned off using a brush and an air blower. Cyanoacrylate was poured onto the model and then placed in an oven at 70 °C for 15 minutes. The part was dipped in paraffin wax for 5 seconds and then placed in an oven at 70 °C for 15 minutes. |
| E | A breakaway support strategy was utilized to adhere the model to the build platform. The model was oriented so that no support material was present in the area subsequently being probed by the ultrasound. The layer thickness was set to 100 microns. | The breakaway support material was manually removed. |
| F | Supports were added to adhere the model to the build platform. The model was oriented so that no support material was present in the area subsequently being probed by the ultrasound. The layer thickness was set to 100 microns. | The support material was manually removed using wire cutters. The part was agitated for 30 seconds in 99% isopropyl alcohol then soaked for 3 minutes in one bath then 3 minutes again in a second isopropyl alcohol bath. The parts were dried with compressed air and cured at 60 °C for 30 minutes and exposed to 405 nm light. |
| G | Supports were added to adhere the model to the build platform. The model was oriented so that no support material was present in the area subsequently being probed by the ultrasound. The layer thickness was set to 100 microns. | The model was placed in an ultrasonic isopropyl alcohol wash basin for 15 minutes to soften the supports. The supports were manually removed and then the model was placed in a second clean isopropyl alcohol ultrasonic bath for 15 minutes. The part was air dried for 15 minutes and then placed in a heated chamber at 60 °C for 30 minutes and exposed to 405 nm light. |
| H | A breakaway support strategy was utilized to adhere the model to the build platform. The model was oriented so that no support material was present in the area subsequently being probed by the ultrasound. The layer thickness was set to 100 microns. | The breakaway support material was manually removed. |
| I | A breakaway support strategy was utilized to adhere the model to the build platform. The model was oriented so that no support material was present in the area subsequently being probed by the ultrasound. The layer thickness was set to 100 microns. | The breakaway support material was manually removed. |
| J | Supports were added to adhere the model to the build platform. The model was oriented so that no support material was present in the area subsequently being probed by the ultrasound. The layer thickness was set to 100 microns. | The support material was manually removed using wire cutters. The part was agitated for 30 seconds in 99 % isopropyl alcohol then soaked for 5 minutes in one bath then 5 minutes again in a second isopropyl alcohol bath. The parts were dried with compressed air and cured at 60 °C for 30 minutes and exposed to 405 nm light. |
| K | Wax support was utilized to adhere the model to the build platform. The model was oriented so that no support material was present in the area subsequently being probed by the ultrasound. This produced a glossy surface that was examined by the ultrasound. The layer thickness was set to 100 microns. | The wax support material was removed by placing the model in an oven at 70 °C for 5 hours. The model was then dipped in 40 °C mineral oil for 30 seconds. The model was then washed in 30 °C soapy water for 5 minutes and then rinsed off with tap water. |
| L | A breakaway support strategy was utilized to adhere the model to the build platform. The model was oriented so that no support material was present in the area subsequently being probed by the ultrasound. The layer thickness was set to 100 microns. | The breakaway support material was manually removed. |
| M | A breakaway support strategy was utilized to adhere the model to the build platform. The model was oriented so that no support material was present in the area subsequently being probed by the ultrasound. The layer thickness was set to 100 microns. | The breakaway support material was manually removed. |

^a^ All models were printed at a shell thickness of 5 mm.
