# Supplementary material for: Various 3D printed materials mimic bone ultrasonographically: 3D printed models of the equine cervical articular process joints as a simulator for ultrasound guided intra-articular injections
Source: PLoS One. 2019 Aug 6;14(8):e0220332. doi: 10.1371/journal.pone.0220332 (PMC6684155; doi:10.1371/journal.pone.0220332)
Supplement: S1 File — (DOCX) [file pone.0220332.s002.docx]

**Survey assessing the three dimensional printed models ultrasonographic features as compared to the gold standard**

**Please circle one of each of the following:**

**What is your position?** Resident / Faculty

**How many years of US experience do you have?** 1-2 years/3-5 years/6-10 year/10+ years

Participant ID#______________ Model #______________

Please state your level of agreement for the following statements by marking a vertical line on a scale between strongly disagree and strongly agree.

**1. Ultrasonographic features of the superficial wall (cortex) of the model are comparable to the gold standard.**

Strongly disagree Strongly agree

**2. The reverberation artifact noted at the surface of the model is comparable to the gold standard.**

Strongly disagree Strongly agree

**3. The articular process joint space size and visibility of the model is comparable to the gold standard.**

Strongly disagree Strongly agree

**4. Internal structures/echoes are noted in the model, unlike the gold standard.**

Strongly disagree Strongly agree

**5. Artifacts are produced by the model that would prevent its use as a training model, compared to the gold standard.**

Strongly disagree Strongly agree

**6. The model would be an acceptable replacement to the gold standard as a training tool for ultrasound guided procedures involving bones/joints.**

Strongly disagree Strongly agree
