# Supplementary material for: Various 3D printed materials mimic bone ultrasonographically: 3D printed models of the equine cervical articular process joints as a simulator for ultrasound guided intra-articular injections
Source: PLoS One. 2019 Aug 6;14(8):e0220332. doi: 10.1371/journal.pone.0220332 (PMC6684155; doi:10.1371/journal.pone.0220332)

**Development of 3D printed models of the sacroiliac, hip, and cervical articular process joints for ultrasound guided joint injection training of equine practitioners**

**Stage 1 – Testing of 3D printed models of cervical articular facet in water**

**Directions**

1. If not already done, please download the consent form*, sign and email it.
2. You are asked to download and look at 15 ultrasound videos (14 models, 1 gold standard). The videos are in MOVIE format (total of 185.2MB, download time of approximately 2 minutes).
   - The order in which videos are reviewed is randomized for each participant. Please follow the order provided in the word document sent to your email address. You may go back and forth between videos.
3. Upon watching all videos, you will answer a questionnaire of 6 questions for each model, for a total of 14 questionnaires/forms.
   - Please complete the forms digitally to help us standardize and anonymize the process.
   - For each question, you are asked to draw a vertical line on a scale. This can be done with «Preview» for MAC users.
   - Carefully identify each form with both the letter attributed to the models and your participant ID. The later should be a unique 4 digit number that you will easily remember (e.g. day and month of father's birthday).
4. Results will be anonymized by a third party before being sent to us. Due to time constraints, we would appreciate if all forms could be returned by Wednesday night (05/03/18). The whole process should take approximately 30-40 minutes.

* The consent form, 15 videos and questionnaire can be found in drop box. Please find link sent to your email address.

** Below are examples of features assessed in the questionnaire.

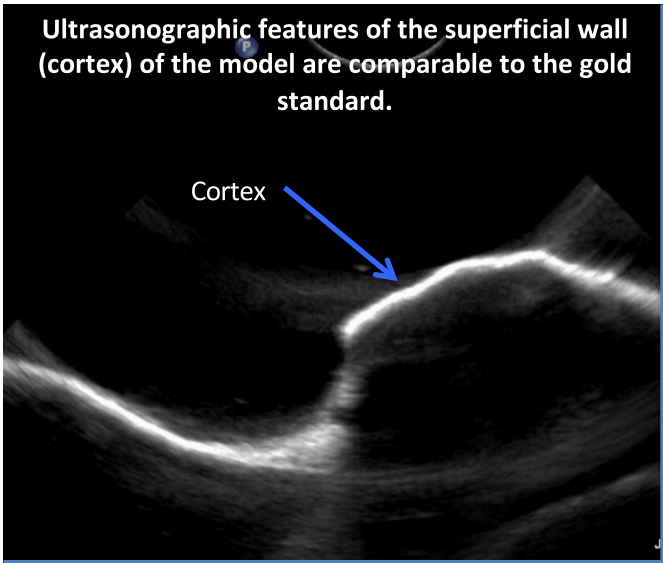


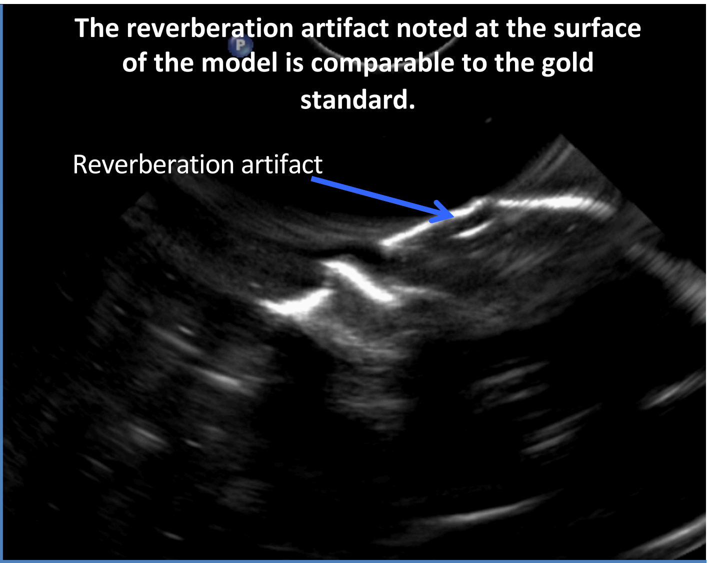


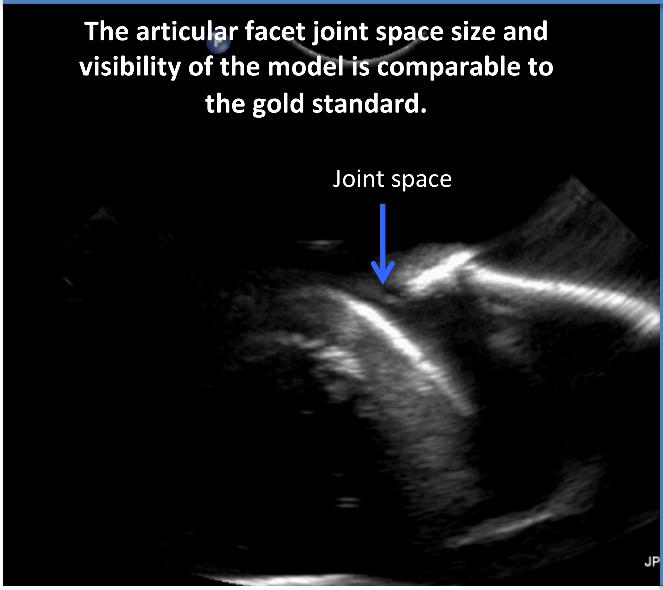


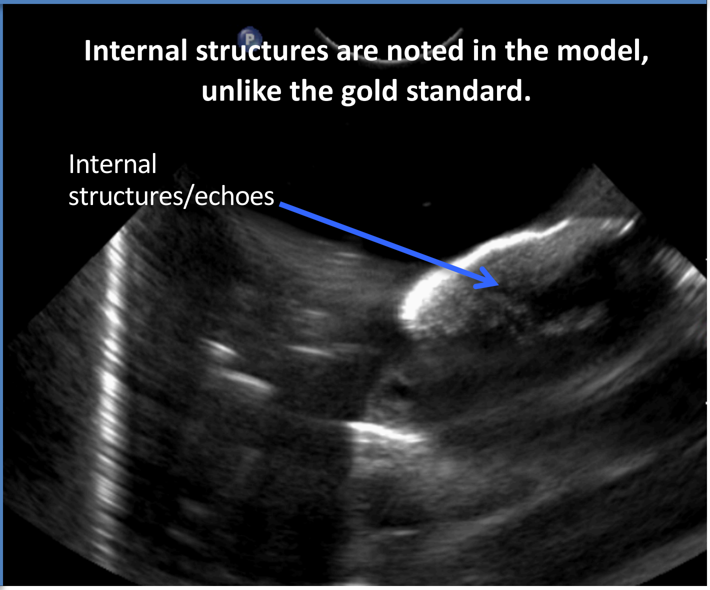

Supplement: S3 File — (PDF) [file pone.0220332.s004.pdf]
